# Humeral stress fracture in a female CrossFit athlete: a case report

**DOI:** 10.1186/s12891-019-2532-1

**Published:** 2019-04-09

**Authors:** Ivan R. B. Godoy, Eduardo A. Malavolta, Jan Stefan Lundberg, Jader J. da Silva, Abdalla Skaf

**Affiliations:** 1 Department of Radiology, Hospital do Coração (HCor) and Teleimagem, Rua Desembargador Eliseu Guilherme, 53, 7th floor, São Paulo, SP CEP 04004-030 Brazil; 2 Department of Orthopedic Surgery, Hospital das Clínicas HCFMUSP, Faculdade de Medicina, Universidade de São Paulo, São Paulo, SP Brazil; 3 Hospital do Coração (HCor), São Paulo, SP Brazil

**Keywords:** Shoulder, Stress fracture, CrossFit

## Abstract

**Background:**

Humeral stress fractures are rare injuries usually related to sports practice and joint overload without a direct trauma. A proximal humeral stress fracture has never been reported in a CrossFit athlete.

**Case presentation:**

We report a stress fracture in the humerus of a 22-year-old woman after intense CrossFit training. Patient’s previous medical history included amenorrhea and reduced Vitamin D levels. The patient was treated conservatively and resumed CrossFit training after she was advised not to until follow up imaging.

**Conclusions:**

We present the MRI features of the case and emphasize the difficulties in diagnosis due to multiple possible causes of shoulder pain in a CrossFit athlete and by negative findings on early radiographs. Hormonal variations, Vitamin D insufficiency and the patient’s attitude towards exercise were important factors that contributed for the stress injury after weight-lifting in CrossFit.

## Background

Stress fractures are rare injuries characterized by the presence of a transverse, oblique or longitudinal bone fracture line without a direct trauma. Approximately 9% of stress fractures occur in the upper extremity [1]. The distal humeral shaft is usually affected in throwing sports such as baseball and lacrosse [2, 3], while proximal humeral stress fractures are related to weight lifting sports [4]. CrossFit was founded in the early 2000s in the United States and has seen a huge growth in popularity around the world [5]. CrossFit is based on varied high intensity functional movements, associated with weightlifting [6]. Recent studies reported significant improvement of strength and fitness of CrossFit athletes, including greater maximal aerobic capacity and body composition [6, 7]. Military studies reported significant improvements in work capacity after CrossFit conditioning program [6]. However, authors noticed an apparent disproportionate musculoskeletal injury risk, especially for novice participants [7].

Recent studies reported injury prevalence and rates with CrossFit practice [8, 9]. Those studies reported an injury prevalence of 73–75% of the participants, with rates of 3.1 to 2.4 per 1000 h trained, respectively [8, 9]. Upper extremity, mostly shoulder, was the most frequently injured body region [8, 9]. Although shoulder injuries often occur during weightlifting and resistance training [10], it is unclear if proper coaching, a beginner’s program, athlete’s BMI and number of rest days during CrossFit training week may reduce shoulder injury rates [10]. To present days, reported injury rates are similar to other weightlifting activities [10–15]. However, future studies could identify specific CrossFit training variables and demographic particularities that might lead to injuries [10, 16]. The shoulder is the most injured joint in CrossFit practice, and 23 to 31% of the athletes have reported shoulder pain or any kind of shoulder injury [8]. Previous studies showed upper extremities lesions related to CrossFit, such as rhabdomyolisis after pull-ups [17] and traumatic tear of the latissimus dorsi myotendinous junction [18]. Up to this date, we are unaware of any reported case in the current literature of shoulder lesion in CrossFit with bone involvement such as this proximal humeral stress fracture in a young woman. We describe the radiographic and MR images of a stress fracture affecting the patient’s right proximal humeral diaphysis and associated findings. We also highlight the importance of accurate imaging evaluation, to exclude other causes of shoulder pain. This report describing a novel finding of a bone stress injury in a CrossFit athlete and contributes to better understanding of possible upper extremity lesions during weight-lifting in this sport modality.

## Case presentation

A 22-year-old woman presented with intense pain in the shoulders that began 2 weeks after injury during a CrossFit competition, especially on the right side, without edema or reduced range of motion. She started the CrossFit practice routine 2 months before the injury. The patient trained CrossFit three times a week without any other sport activity on the remaining week days. Olympic weightlifting (overhead movement) was described as the last activity before the acute pain started. The initial overhead load was 55 pounds and was part of a “novice” training routine with reported adequate technique. One week prior to the CrossFit competition the patient was oriented to increase the Olympic weightlifting load to 75 pounds as a requirement for competition. Repetitions were set to her own limitation. After the injury, she stopped her exercises and applied ice to the shoulders and upper arms. During the following days, she experienced continuous pain and sought medical attention. Physical examination did not show any reduction of passive and active arcs of movements. Also, Jobe and Bear Hug tests were negative. There was only a mild ligamentous laxity and 10 degrees of elbow hyperextension. Her medical history did not include medications, prior fractures, neoplastic disease or prior surgery on the upper extremities. Also, she did not have history of anabolic or glucocorticoid steroid use. The patient’s height was 1.53 m and weighted 54 Kg, with a BMI of 22.9 and was physically active for more than 3 years. She has never had CrossFit lessons or prior training. Before she started CrossFit practice, the patient used to swim three times a week in college, but did not participate in competitions. The patient had an episode of amenorrhea 3 years ago, considered to be due to weight loss, probably due to intense training and reduced caloric intake. Laboratory tests and densitometry were performed in the same period of the imaging studies and showed normal mineral bone density and Vitamin D insufficiency (serum Vitamin D of 24.8 ng/mL). During the two initial weeks of the patient’s bilateral upper limb pain, she attributed the symptoms to “overtraining” but she sought medical attention when the pain became progressively worse. The patient did not refer any specific diet, nutritional supplementation or reduced caloric intake during the time of the shoulder lesion.

Radiographs of the shoulders were performed 1 week after onset of symptoms.

**Fig. 1:**
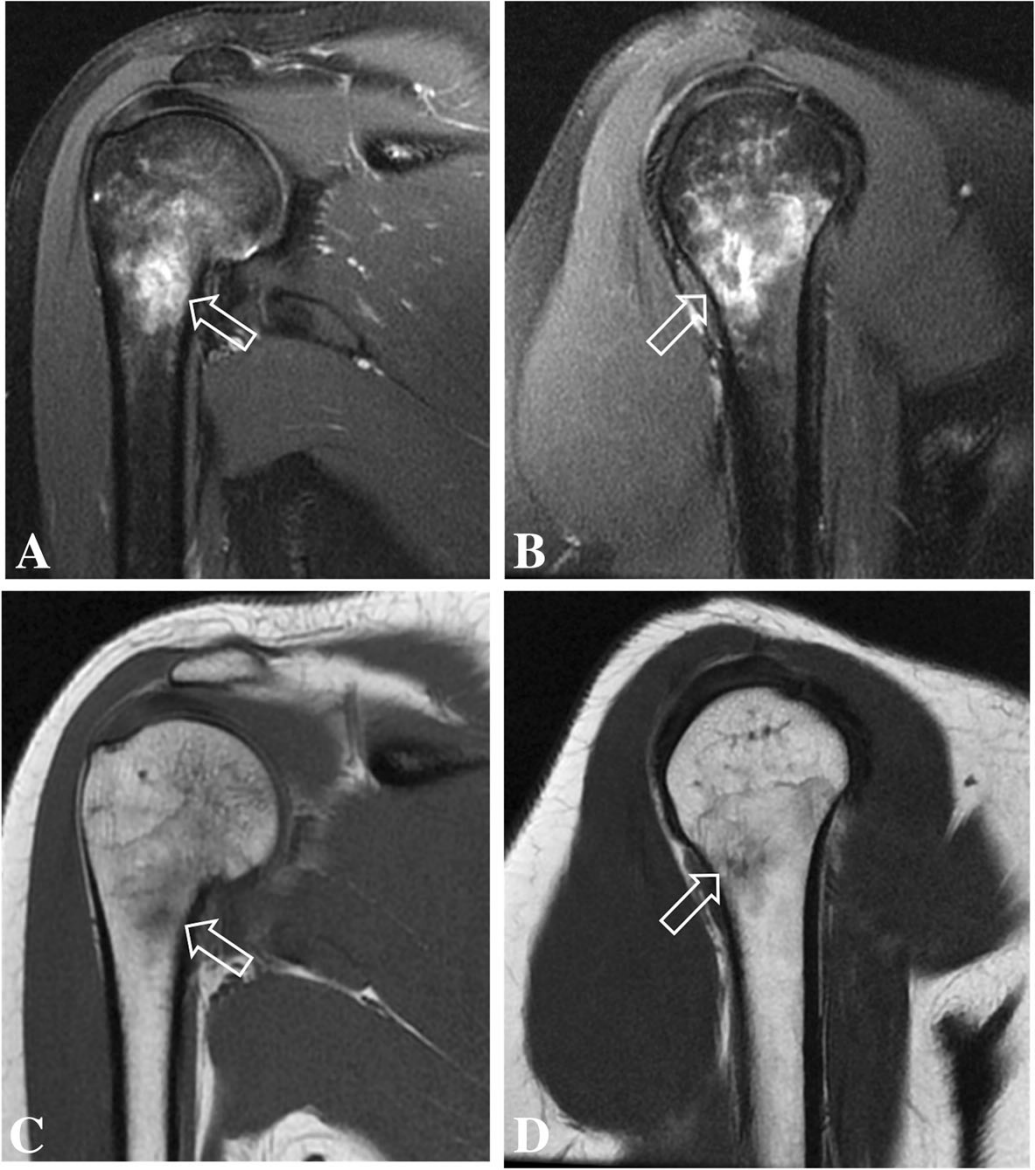
Coronal (**a**) and sagittal (**b**) T2-weighted fat-suppressed MR images and coronal (**c**) and sagittal (**d**) T1-weighted MR images of the right shoulder showing intense bone marrow edema of the proximal humerus, with a small incomplete transverse fracture line (open arrow)

**Fig. 2:**
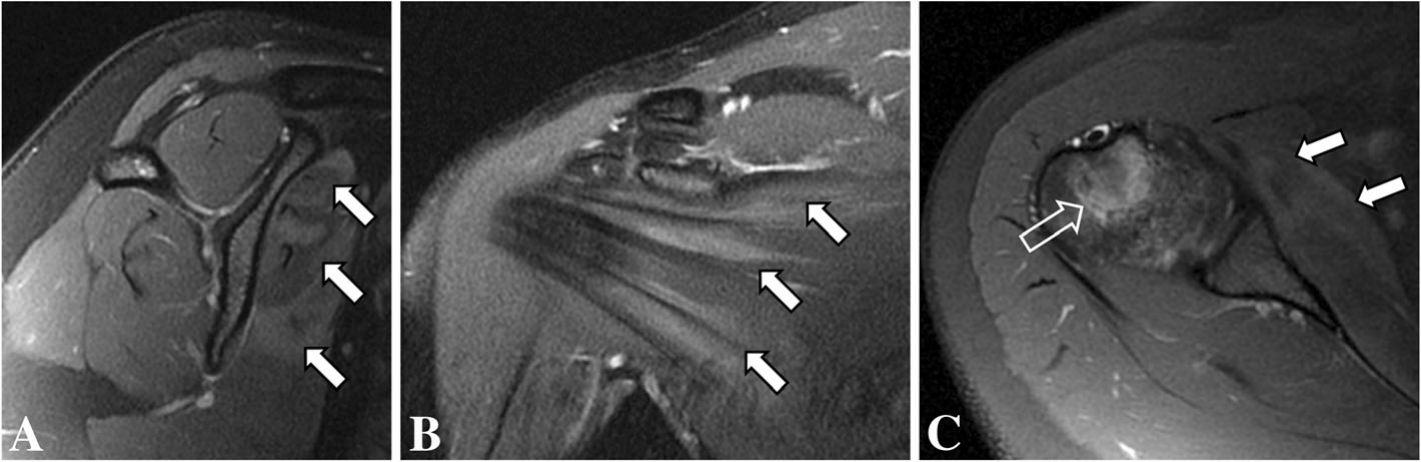
Sagittal (**a**), coronal (**b**) and axial (**c**) T2-weighted fat-suppressed MR images of the right shoulder showing intense bone marrow edema of the humerus (open arrow), with edema of the subscapularis muscle (arrows)

**Fig. 3:**
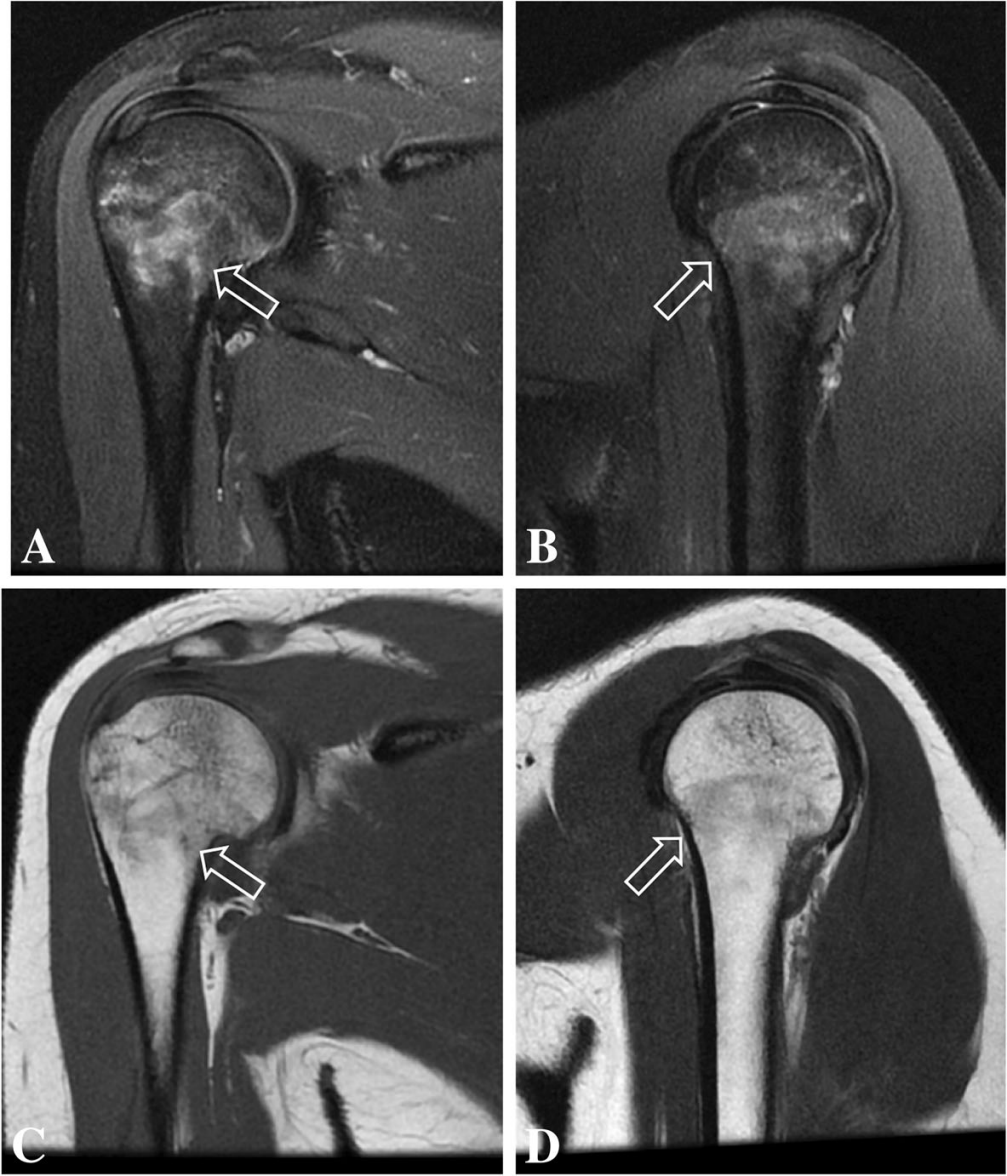
Coronal (**a**) and sagittal (**b**) T2-weighted fat-suppressed MR images and coronal (**c**) and sagittal (**d**) T1-weighted MR images of the left shoulder showing intense bone marrow edema of the proximal humerus, without fracture lines or periosteal reaction (open arrow)

**Fig. 4:**
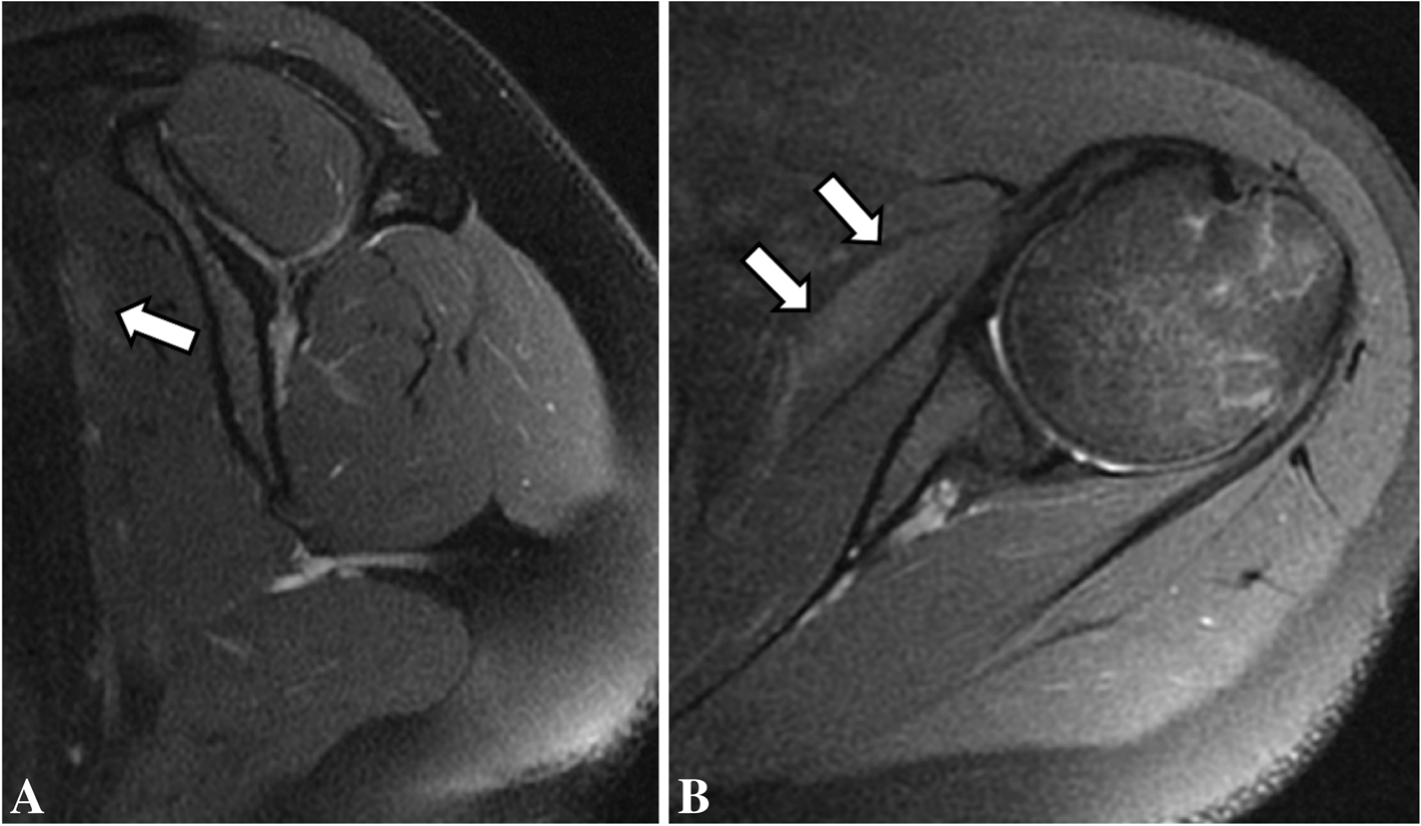
Sagittal (**a**) and axial (**b**) T2-weighted fat-suppressed MR images showing mild edema of the subscapularis muscle (arrows)

These were interpreted as normal, showing no evidence of bone lesion, fracture line, arthritis, or periosteal bone formation. However, small and incomplete fracture lines may not be seen on early radiographs. At that time, the patient was presumed to have bilateral impingement syndrome. MRI of the shoulders were performed 2 weeks after the onset of symptoms (Figs. 1 and 2) to exclude other causes of shoulder pain such as rotator cuff injuries, bursitis, bone marrow edema and small fractures The MR images of the right shoulder showed extensive bone marrow edema throughout the right humeral medullary cavity. In addition to marrow edema, there was an incomplete transverse fracture line adjacent to the medial cortex of the surgical neck and mild soft tissue edema (Fig. 1). Further, there was an intense edema of the subscapularis muscle, with scattered pattern suggesting overload injury probably related to delayed onset of muscle soreness (DOMS), without tears. (Figs. 1 and 2) On the left side the findings were less prominent, and similarly showed bone marrow edema of the proximal humerus, without a defined fracture line, considered to be the result of joint / bone overload (Fig. 3). Additionally, there was a mild edema of the subscapularis muscle, without tears (Fig. 4).

The patient was treated nonoperatively with activity modification, without immobilization. She received oral non-steroid anti-inflammatory drugs for 5 days and 20 physiotherapy sessions for analgesia, stretching and strengthening of the rotator cuff and deltoid. Also, the patient had vitamin D reposition with 7000 IU twice a week in the first month, with 7000 IU weekly as maintenance. One month later the patient was asymptomatic and 3 months later she resumed CrossFit training against medical advice not to return to sports without follow-up imaging.

**Fig. 5:**
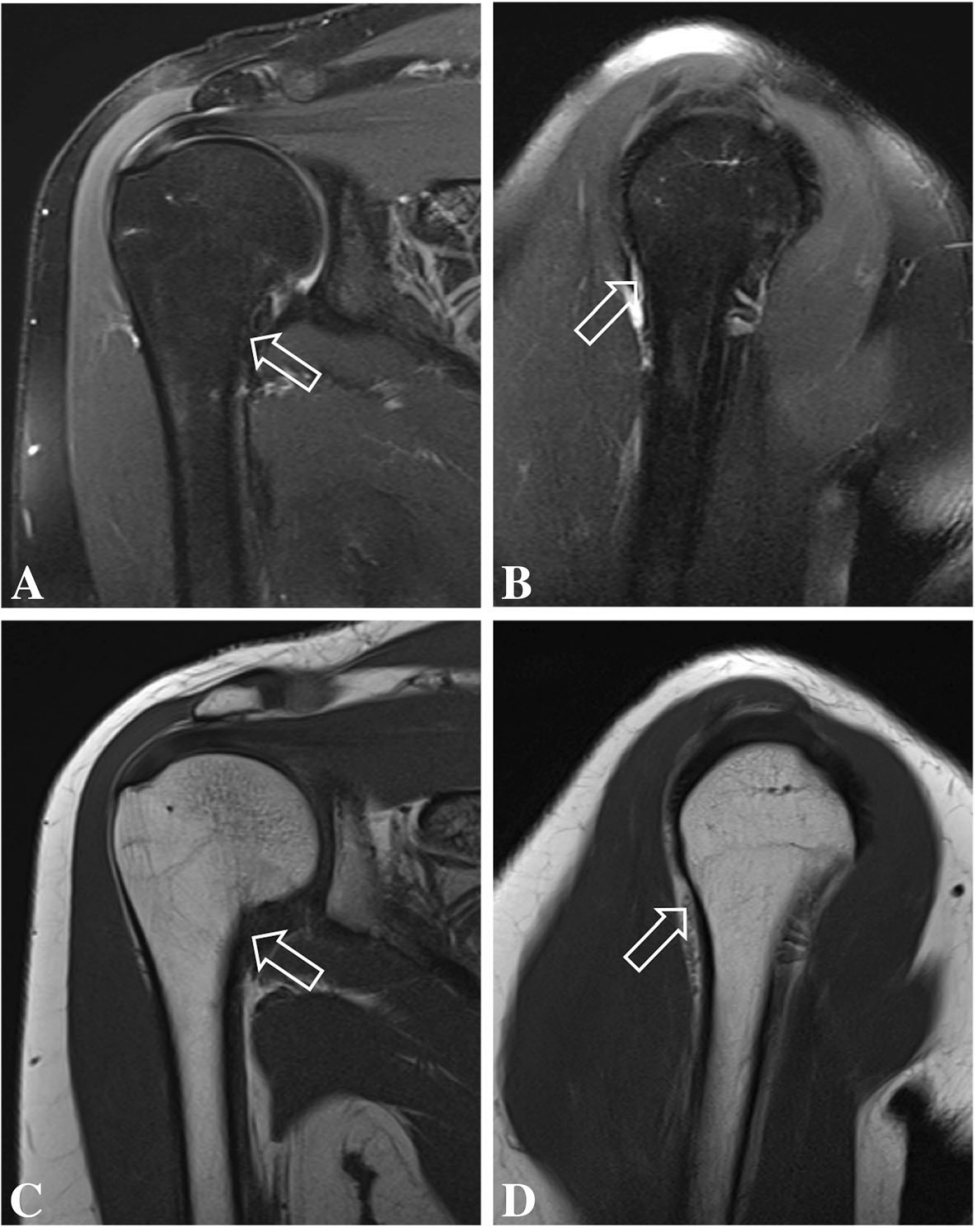
Coronal (**a**) and sagittal (**b**) T2-weighted fat-suppressed MR images and coronal (**c**) and sagittal (**d**) T1-weighted follow-up MR images of the right shoulder show complete resolution of bone marrow edema of the proximal humerus, with consolidation of the transverse fracture line (open arrow)

**Fig. 6:**
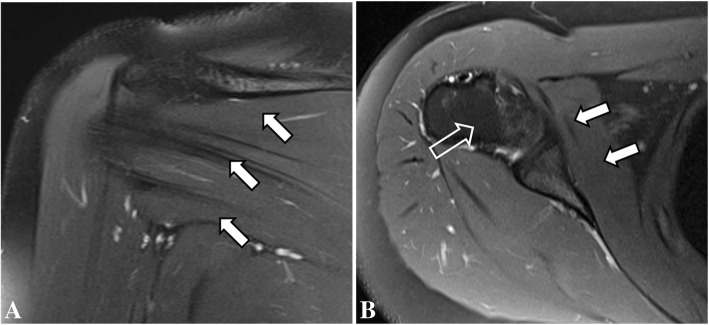
Coronal (**a**) and axial (**b**) T2-weighted fat-suppressed follow-up MR images of the right shoulder showing complete resolution of the bone marrow edema of the humerus (open arrow) and of the subscapularis muscle edema (arrows)

**Fig. 7:**
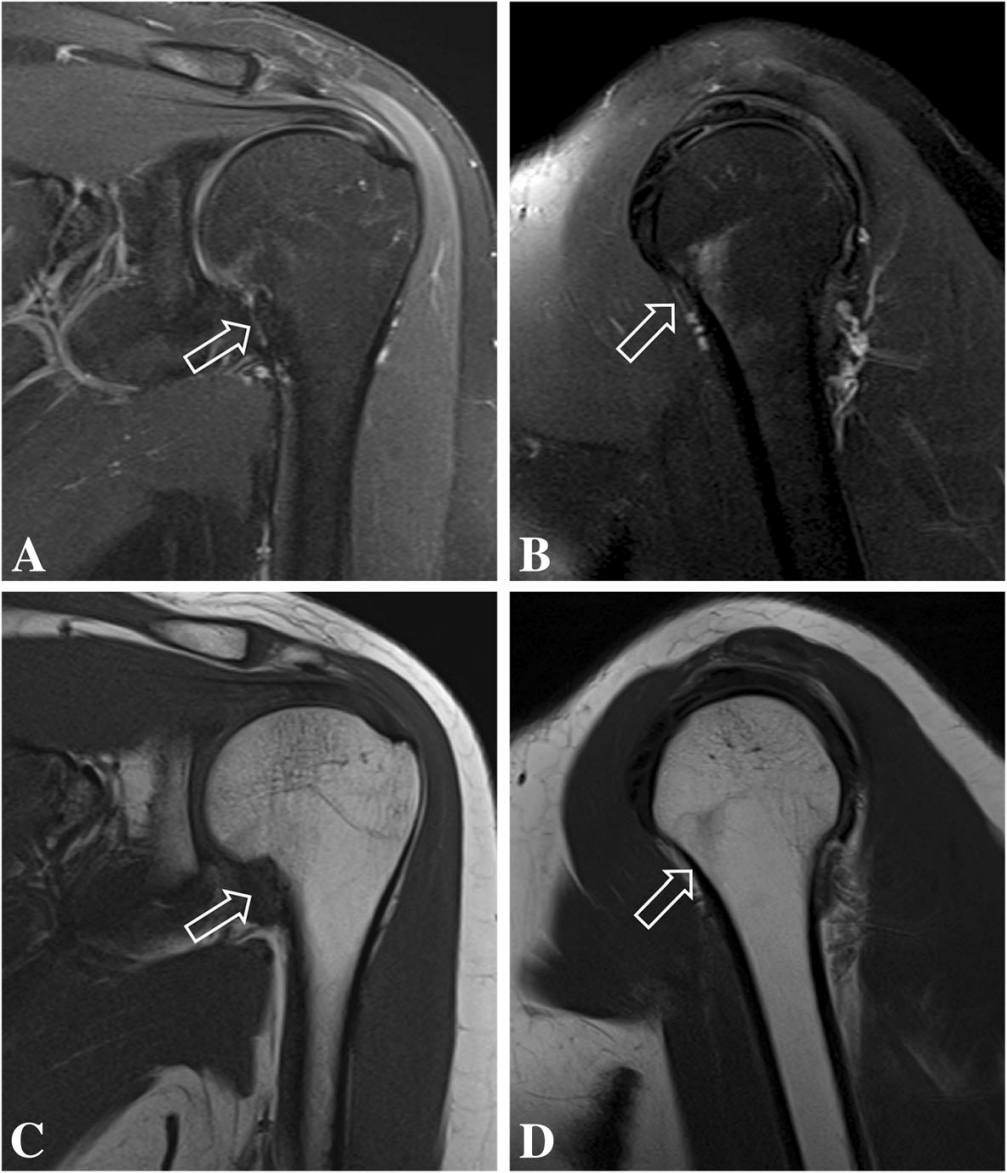
Coronal (**a**) and sagittal (**b**) T2-weighted fat-suppressed follow-up MR images and coronal (**c**) and sagittal (**d**) T1-weighted MR images of the left shoulder showing resolution of the bone marrow edema of the proximal humerus (open arrow)

**Fig. 8:**
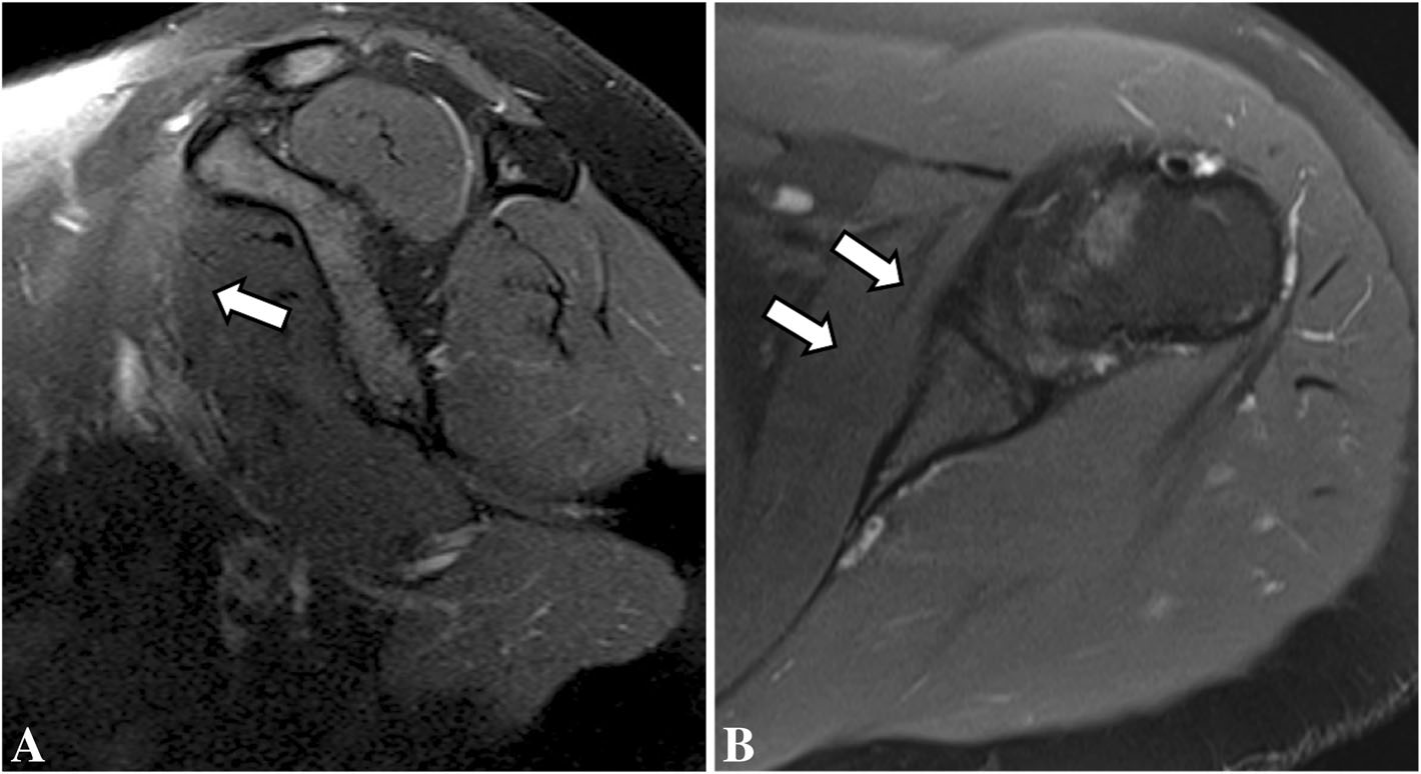
Sagittal (**a**) and axial (**b**) T2-weighted fat-suppressed follow-up MR images showing resolution of the subscapularis muscle edema (arrows)

Follow-up MRI was done 4 months after the initial studies and showed complete resolution of bone edema and consolidation of the transverse fracture line on the right proximal humeral diaphysis. Also, there was complete resolution of bone marrow edema on the left humerus, and no visible bilateral subscapularis muscle edema or tear (Figs. 5, 6, 7, 8). After 6 months of follow-up the patient was asymptomatic.

## Discussion and conclusions

Humeral stress fractures have been described in sports activities with upper extremities overuse such as tennis [19], baseball [2, 20], weight lifters [9, 14], arm wrestlers [21, 22] or even in military activities including grenade throwing [23]. There is a potential risk of stress to became complete fractures with a possible surgical treatment and longer time return to sports practice. [2, 20–23].

Radiographs of stress fractures are frequently normal [24, 25] therefore, MRI is paramount to identify small fracture lines and exclude other possible causes of bone and joint pain. The MRI features of a stress fractures can be nonspecific, varying from a prominent bone marrow edema with or without periosteal reaction [24, 25] to a distinct fracture line. Without the adequate clinical setting, these findings may be misinterpreted as evidence of infection or neoplasia [26–28]. Stress fractures generally presents with indolent pain for several weeks, usually affecting two groups of patients: athletes and patients with osteoporosis (most commonly postmenopausal women) [29, 30]. In this reported, the stress fracture of the proximal humeral shaft was probably related to a bone overload in a young woman with prior history of amenorrhea, reduced Vitamin D levels associated with intense CrossFit training. The patient of this case report had low serum levels of vitamin D at the time of the injury. Studies reported that vitamin D deficiency may lead to stress fractures [2, 4]. A military randomized, double-blind, placebo-controlled study observed a lower rate of stress fractures in female recruits with calcium and vitamin D supplementation, suggesting a protector factor of normal serum vitamin D levels [23]. After 3 months of vitamin D supplementation, our patient achieved normal serum levels (Follow-up Vitamin D level: 32.0 ng/mL).

The patient also had a previous episode of temporary amenorrhea, considered to be due to excessive training and dietary approaches with reduced caloric intake. Currently, there is a concept suggesting the between reduced dietary intake and amenorrhea [19]. If caloric intake is too low, production of hormones such as estrogen and progesterone may not be enough produced to induce regular menstruation [19]. There have been reports of increased rates of stress fractures in female athletes and military with risk factors as low body mass index (BMI), menstrual irregularity, reduced caloric intake, and decreased bone mineral density, compared with males [20, 28].

A recent CrossFit systematic review and meta-analysis demonstrated an initial evidence of higher levels of sense of community, satisfaction, and motivation among CrossFit participants [31]. The motivational characteristics of the CrossFit practice is focused on leading the individual to achieve the best performance possible. In this sport modality there is a 5% prevalence of exercise addiction reported [32]. This behavior is associated with a tendency to exercise despite injuries, feelings of guilt when unable to exercise, passion turning into obsession, and taking medication to be able to practice [32]. Our patient returned to CrossFit after she was advised not to until complete resolution of the symptoms and follow up imaging. This reveals her high level of engagement and may raise the suspicion for exercise addiction. However, the patient did not report body dysmorphia or prior negative attitudes toward exercise. A mental status investigation in cases similar to ours is advisable to prevent re-injuries that could lead to delayed return to sport [32].

In this report, a proximal humeral stress fracture was described in a young woman, with contralateral humeral bone marrow edema probably due to mechanical overload during CrossFit practice. Also, there was bilateral subscapularis muscle edema, related to DOMS and was considered to be a contributor to bilateral shoulder pain, without tears. The imaging findings of shoulder MRI and negative radiographs were key to diagnosis this condition and excluded other possible causes of upper extremity pain, such as rotator cuff injury. We considered that female gender, and vitamin D insufficiency associated with extreme training routine were possible predisposing factors for this uncommon proximal humeral stress fracture. Analgesia and joint rest may lead to resolution of symptoms and fracture healing. If necessary, vitamin D supplementation and monitoring, as well as bone mineral density assessment should be recommended, to prevent further fractures. Follow-up radiographs and MRI are useful to monitor bone consolidation and possible complications. Further studies are necessary to define potential risks to shoulder injury in CrossFit training, however the current literature suggests similar risks as other weightlifting sport activities. Although mental benefits of CrossFit are evident such as increased levels of satisfaction and motivation, careful training program selection and progression should be specific to each participant to avoid injuries. Also, addiction-like behavior should be monitored for best practice.

## Abbreviations

BMI: Body mass index
DOMS: Delayed onset of muscle soreness
MRI: Magnetic Resonance imaging
