# Correction to: Humeral stress fracture in a female CrossFit athlete: a case report

**DOI:** 10.1186/s12891-019-2674-1

**Published:** 2019-06-18

**Authors:** Ivan R. B. Godoy, Eduardo A. Malavolta, Jan Stefan Lundberg, Jader J. da Silva, Abdalla Skaf

**Affiliations:** 1 Department of Radiology, Hospital do Coração (HCor) and Teleimagem, Rua Desembargador Eliseu Guilherme, 53, 7th floor, São Paulo, SP CEP 04004-030 Brazil; 2 Department of Orthopedic Surgery Hospital das Clínicas HCFMUSP, Faculdade de Medicina, Universidade de São Paulo, São Paulo, SP Brazil; 3 Hospital do Coração (HCor), São Paulo, SP Brazil

**Correction to: BMC Musculoskelet Disord**

**https://doi.org/10.1186/s12891-019-2532-1**

It has been brought to our attention that the article [1] in reference [7] *Smith MM, Sommer AJ, Starkoff BE, Devor ST. Crossfit-based high-intensity power training improves maximal aerobic fitness and body composition. J Strength Cond Res. 2013;27(11):3159–72* was retracted in October 2017. At the time of the submission of the manuscript the authors were not aware of the retraction and legal implications related to this reference. Therefore, the authors do not support the findings of the retracted article such as that CrossFit has *“an apparent disproportionate musculoskeletal injury risk, especially for novice participants”.* The overall conclusions of the case report are not affected by this erratum.

## References

[1] Godoy IRB (2019). Humeral stress fracture in a female CrossFit athlete: a case report. BMC Musculoskelet Disord.

